# Exosomal HMGB1 derived from hypoxia‐conditioned bone marrow mesenchymal stem cells increases angiogenesis via the JNK/HIF‐1α pathway

**DOI:** 10.1002/2211-5463.13142

**Published:** 2021-04-01

**Authors:** Wenling Gao, Ronghan He, Jianhua Ren, Wenhui Zhang, Kun Wang, Lei Zhu, Tangzhao Liang

**Affiliations:** ^1^ Department of Orthodontics Hospital of Stomatology Sun Yat‐sen University Guangzhou China; ^2^ Department of Orthopaedic Surgery the Third Affiliated Hospital of Sun Yat‐sen University Guangzhou China; ^3^ Department of Plastic and Reconstructive Surgery the Third Affiliated Hospital of Sun Yat‐sen University Guangzhou China

**Keywords:** angiogenesis, bone marrow mesenchymal stem cells, exosome, HMGB1, hypoxia

## Abstract

Mesenchymal stem cells (MSCs) have been described to induce angiogenesis in various tissues and have been used for the development of novel cell‐based therapies. Increasing evidence suggests that MSCs execute their paracrine function via the secretion of exosomes, especially under hypoxic conditions. However, the mechanisms by which MSC‐derived exosomes secreted under hypoxia enhance angiogenesis still remain unclear. To study exosome physiology under hypoxic or normoxic conditions, we isolated exosomes from bone marrow mesenchymal stem cells (BMSCs). Furthermore, we detected the uptake of exosomes by human umbilical vein endothelial cells (HUVECs) by immunofluorescence staining. In addition, we determined the effects of exosomes on cell viability, migration and tube formation in HUVECs by Cell Counting Kit‐8, migration and tube formation assays, respectively. We examined the expression of key proteins related to exosome‐induced angiogenesis by BMSCs cultured under hypoxic conditions by western blot. Exosomes released by BMSCs cultured under hypoxic conditions enhanced cell proliferation, migration and angiogenesis of HUVECs. H**y**poxia induced the expression of high mobility group box 1 protein (HMGB1) in BMSC‐derived exosomes, and silencing of HMGB1 abolished the angiogenic effect in HUVECs. Furthermore, exosomal HMGB1 activated the JNK signaling pathway and induced hypoxia‐inducible factor‐1α/vascular endothelial growth factor expression, consequently enhancing angiogenesis in HUVECs. Our data reveal that exosomal HMGB1 promotes angiogenesis via JNK/hypoxia‐inducible factor‐1α signaling. Therefore, BMSC exosomes derived under hypoxia may have potential for development of novel treatment strategies for angiogenesis‐related diseases.

## Graphical abstract

To study exosome physiology under hypoxic or normoxic conditions from bone marrow mesenchymal stem cells, we determined the effects of exosomes on cell viability, migration and tube formation in human umbilical vein endothelial cells. Our data reveal that exosomal high mobility group box 1 protein promotes angiogenesis under hypoxic conditions. Bone marrow mesenchymal stem cell exosomes obtained under hypoxic conditions may have potential for development of novel treatment strategies for angiogenesis‐related diseases.

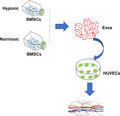

**Abbreviations**
BMSC: bone marrow mesenchymal stem cell
CCK‐8: Cell Counting Kit‐8
HIF: hypoxia‐inducible factor
HMGB1: high mobility group box 1 protein
H‐MSC‐exo: hypoxia‐conditioned BMSC‐derived exosome
HUVEC: human umbilical vein endothelial cell
MSC: mesenchymal stem cell
N‐MSC‐exo: normoxia‐conditioned BMSC‐derived exosome
SD: standard deviation
siRNA: small interfering RNA

Angiogenesis is a complex process that is important for the treatment of various diseases, such as myocardial infarction and severe limb ischemia [1]. Cell‐based therapies, including mesenchymal stem cells (MSCs), have been increasingly used to improve angiogenesis [2]. It has been reported that MSCs accelerate vessel formation via induction of endothelial cell proliferation and migration [3]. Increasing evidence suggests that the positive effect of MSCs on angiogenesis is not because of cell–cell proximity, but rather is attributed to their paracrine activity [4], such as secretion of exosomes, growth factors and cytokines.

Exosomes, 40‐ to 100‐nm extracellular vesicles, can be secreted by nearly all cell types. Exosomes mediate the paracrine effect of MSCs through the transfer of various host bioactive molecules, such as mRNAs, miRNAs, lipids and proteins, to recipient cells and alter their phenotype and function [5]. Multiple research studies have demonstrated that MSC‐secreted exosomes have high potential on angiogenesis. For example, Takeuchi *et al*. [6] have reported that exosomes from bone marrow mesenchymal stem cells (BMSCs) promote bone regeneration via enhancing angiogenesis. Qiu *et al*. [7] have also shown that exosomes released from MSCs enhance angiogenesis via regulating the AKT/endothelial nitric oxide synthase pathway, thus accelerating wound healing. Therefore, MSC‐derived exosomes represent promising targets for angiogenesis‐promoting treatments.

Oxygen content is a critical factor controlling the proliferation and differentiation of MSCs. In contrast with the normoxic condition (21% O_2_) during *in vitro* culture, most MSCs exist in a hypoxic environment (2–8% O_2_) in the body [8]. It has been reported that MSCs cultured under hypoxia show a higher proliferative activity as compared with those cultured under normoxia [9]. Hypoxia treatment can alter the stem cell characteristics of MSCs and influence the secretion of cytokines and growth factors [3]. Importantly, hypoxia‐conditioned BMSC‐derived exosomes (H‐MSC‐exos) display an enhanced angiogenesis‐promoting effect. However, the underlying mechanisms are not fully understood. Hence, in this study, we sought to compare the effects of exosomes derived from hypoxia‐treated and normoxia‐treated MSCs on endothelial cell proliferation, migration and angiogenesis, and explore the potential mechanisms.

## Materials and methods

### Cell culture

Human adipose tissue‐derived stem cells were purchased from Guangzhou Cyagen Biology (Huangpu District, Guangzhou, China). BMSCs were cultured in Dulbecco's modified Eagle's medium (Gibco, Waltham, MA, USA) with 10% FBS (Life Technologies, Gibco, USA) and 1% penicillin–streptomycin (Gibco) in a humidified atmosphere with 5% CO_2_ and 21% O_2_ (normoxia) or 5% O_2_ (hypoxia) at 37 °C [10]. Human umbilical vein endothelial cells (HUVECs) were maintained in endothelial cell growth medium‐2 (EBM‐2) (Lonza, Basel, Switzerland) with EGM‐2 supplement (Lonza).

### Exosome extraction

Medium of BMSCs under hypoxic or normoxic conditions for 6 days as reported previously [10] was collected and applied for exosome extraction. Medium was centrifuged at 3000 ***g*** for 15 min, followed by 20 000 ***g*** for 45 min. After filtering with a 0.22‐μm filter, supernatants were centrifuged at 110 000 ***g*** for 70 min at 4 °C. The exosomes were washed with PBS and centrifuged for another 70 min at 110 000 ***g*** at 4 °C.

### Transmission electron microscopy

BMSC‐derived exosomes resuspended in PBS were dropped on a copper grid and air‐dried. After fixing with 3% glutaraldehyde for 2 h, exosomes were stained with 2% uranyl acetate for 30 s. Exosomes were observed under a transmission electron microscope. The diameter of exosomes was detected by dynamic light scattering.

### Exosome uptake analysis

KH67 Green Fluorescent Cell Linker Kit (Sigma, St. Louis, MO, USA) was used to stain exosomes. Two micrograms of PKH67‐labeled exosomes was added to HUVECs (2 × 10^5^/well) in six‐well plates and incubated for 24 h. Cells were fixed with 4% paraformaldehyde, and the nuclei were stained with DAPI. The location of exosomes in HUVECs was visualized using a confocal microscope (Carl Zeiss, Jena, Germany).

### Western blot analysis

Cells or exosomes were collected and lysed using radioimmunoprecipitation assay. After separating by SDS/PAGE gels, proteins were transferred to poly(vinylidene difluoride) membrane. The membrane was blocked with 5% BSA and incubated with antibodies against CD9 (ab59479; Abcam, Cambridge Science Park, England), CD81 (ab79559; Abcam), TSG101 (ab125011; Abcam), vascular endothelial growth factor (VEGF; 19003‐1‐AP; Proteintech, Rosemont, IL, USA), CD31 (ab28364; Abcam) and glyceraldehyde‐3 phosphate dehydrogenase (#2118; Cell Signaling Technology, Boston, USA). Then the membrane was incubated with the secondary antibody for 1–2 h at room temperature. Chemiluminescence substrate was applicated to detect the protein bands.

### Viability assay

HUVECs were placed in 96‐well plates at a density of 2 × 10^3^ cells/well. The viability of HUVECs was accessed by the Cell Counting Kit‐8 (CCK‐8) assay kit (Dojindo Molecular Technologies, Kumamoto Prefecture, Japan). The absorbance at 450 nm (*A*_450 nm_) was detected using a plate reader every 24 h.

### Migration assay

Transwell migration assay was performed to detect the influence of exosomes on cell migration. HUVECs (1 × 10^5^) in medium without serum were added into the upper chambers of Transwells (Corning, New York, NY, USA), and 600 μL completed medium containing 10 μg·mL^−1^ exosomes was added to the lower chamber. HUVECs were incubated for 24 h at 37 °C, and cells transferred to the bottom of the membrane were fixed and stained with 0.1% crystal violet.

### Tube formation assay

HUVECs (1.5 × 10^4^ cells/well) were seeded in a 24‐well plate precoated with Matrigel (BD Sciences, Franklin Lakes, NJ, USA). Exosomes (10 μg·mL^−1^) derived from BMSCs were added to HUVECs. The number of tubes was counted under a light microscope, and three random fields were used.

### Quantitative RT‐PCR assay

Total RNA was extracted from HUVECs treated with exosomes with TRIzol reagent (Life Science, Waltham, MA, USA). cDNA was reverse transcribed using PrimeScript RT‐PCR kit (TaKaRa, Dalian, China). Quantitative RT‐PCR was conducted according to the SYBR Green manufacturer's instructions (Tiangen Biotech, Beijing, China). The PCR program was as follows: 95 °C for 2 min and then 36 cycles of 95 °C for 15 s, 60 °C for 30 s and 68 °C for 45 s. The experiments were performed in triplicate. Relative gene expression level was normalized to glyceraldehyde‐3 phosphate dehydrogenase and calculated using the $2^{‐\Delta \Delta C_{T}}$ method.

### Small interfering RNA transfection

High mobility group box 1 protein (HMGB1) or control small interfering RNA (siRNA) sequences were synthesized by GenePharma (Hangzhou, China). HUVECs (30–50% confluence) were transfected with siRNA using Lipofectamine 2000 (Thermofisher, Waltham, MA, USA) following the manufacturer's instructions.

### Statistical analysis

All data were expressed as the mean ± standard deviation (SD). Comparisons between two groups were performed with Student’s *t*‐test, while differences among multiple groups were analyzed by one‐way multivariate ANOVA. Statistical significance was considered as *P* < 0.05.

## Results

### Identification of exosomes from BMSCs

**Fig. 1:**
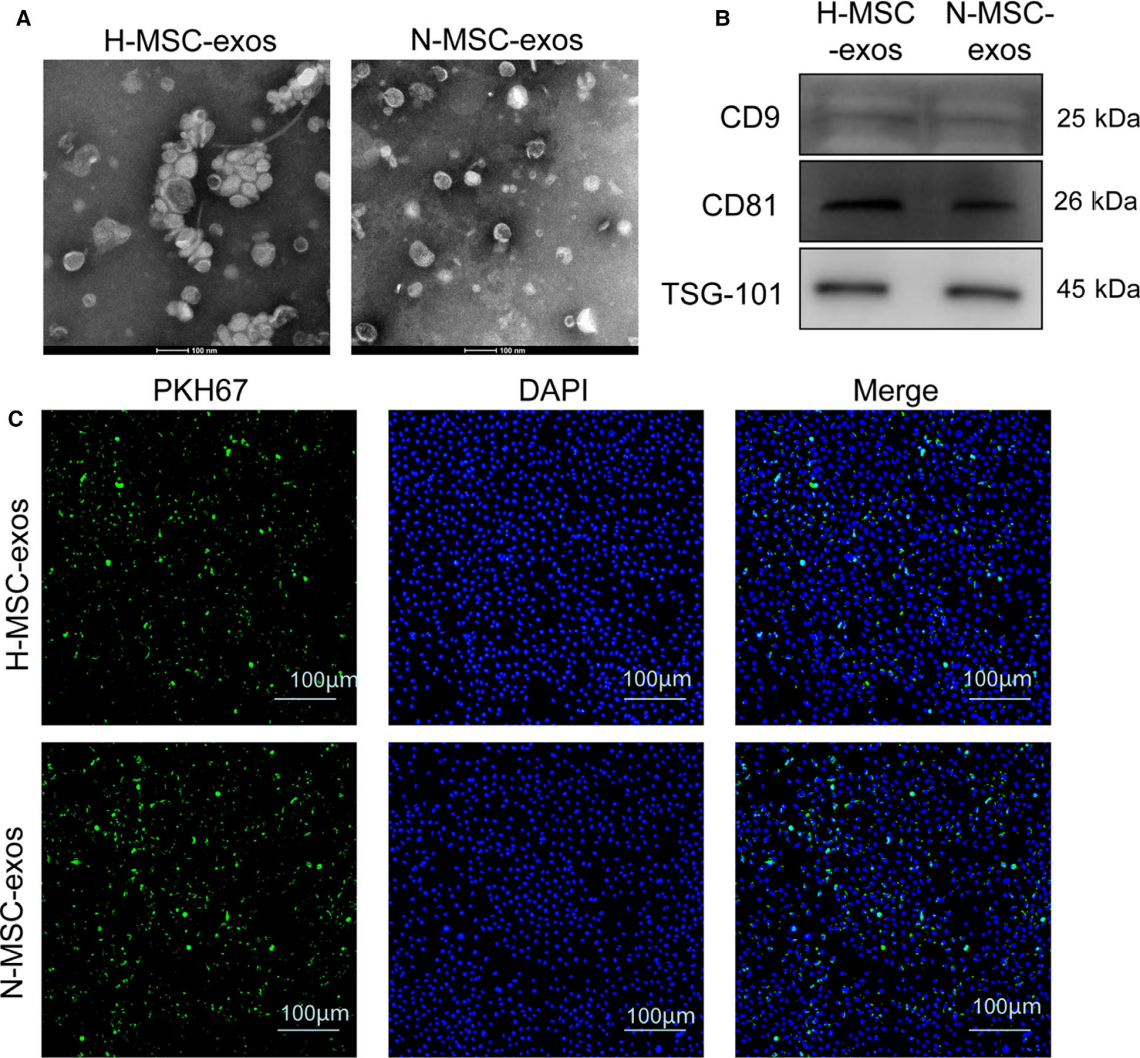
Characterization of BMSC‐derived exosomes. (A). Morphology of H‐MSC‐exos (hypoxia/exos) (left) and N‐MSC‐exos (normoxia/exos) (right) analyzed by transmission electron microscopy. Scale bars: 100 nm. (B) Western blot analysis of CD9 and CD81 expression in exosomes derived from hypoxia or normoxia‐conditioned BMSCs. (C). Uptake of exosomes in HUVECs by immunofluorescence staining. Scale bars: 100 μm.

**Fig. 2:**
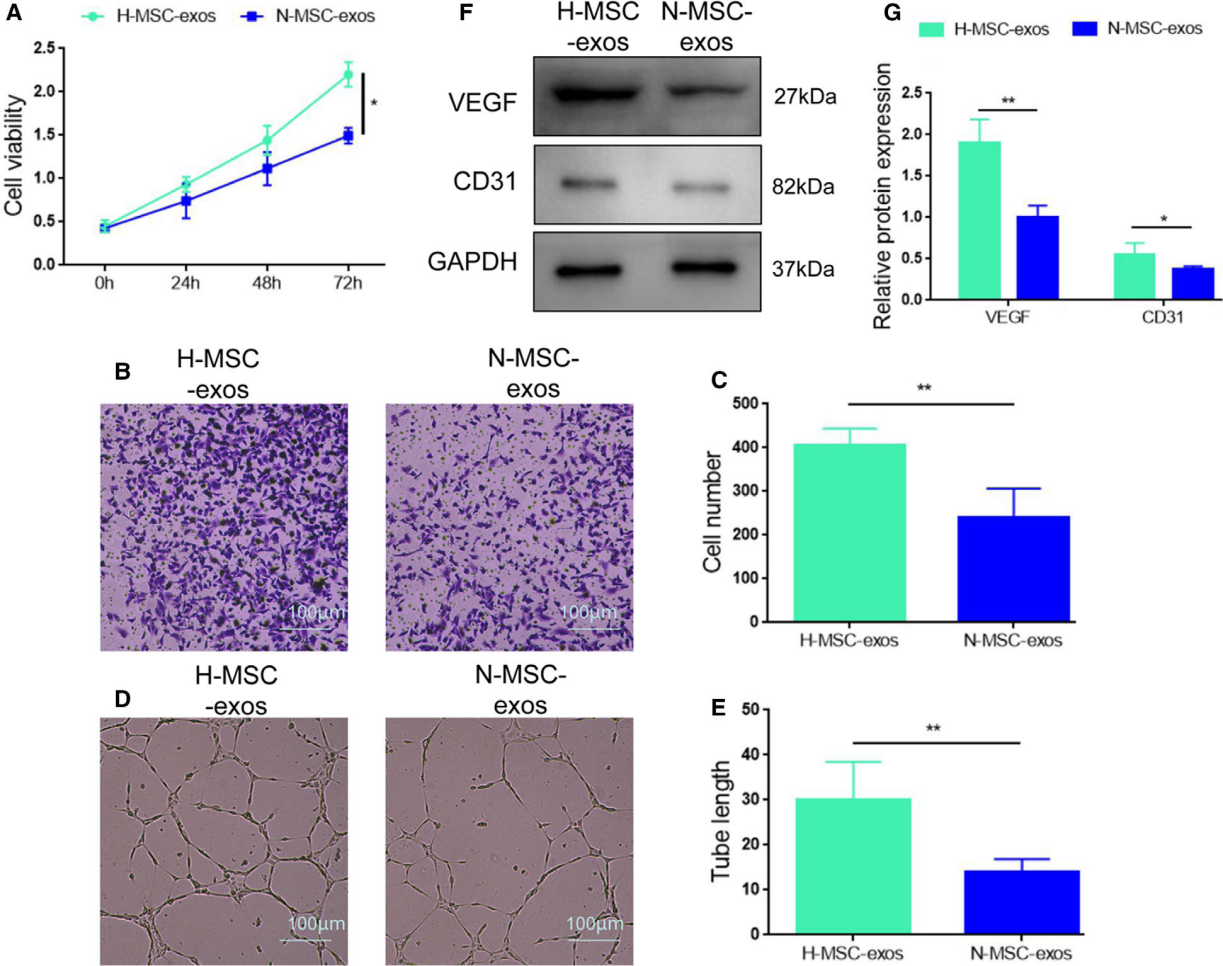
Effects of hypoxic MSC‐derived exosomes on the viability, migration and tube formation of HUVECs. (A). The viability of HUVECs was determined by CCK‐8 assay after treatment with H‐MSC‐exos (hypoxia/exos) or N‐MSC‐exos (normoxia/exos). (B, C) Transwell migration assay in HUVECs after exposure to H‐MSC‐exos or N‐MSC‐exos. (D, E) Tube formation assay in HUVECs treated with H‐MSC‐exos or N‐MSC‐exos. (F, G) Western blot analysis of VEGF and CD31 expression in HUVECs treated with H‐MSC‐exos or N‐MSC‐exos. *P < 0.05, **P < 0.01; *n* = 3. Data represent the mean ± SD of three separate experiments; comparison was performed with Student's *t*‐test. Scale bars: 100 μm.

To investigate the effect of MSC‐derived exosomes on angiogenesis, we isolated exosomes from BMSCs cultured under hypoxic or normoxic conditions via ultracentrifugation. Transmission electron microscopy analysis revealed that a number of spherical exosomes were isolated, and the diameters were 40–100 nm (Fig. 1A). The phenotypes of H‐MSC‐exos and normoxia‐conditioned BMSC‐derived exosomes (N‐MSC‐exos) were similar. To further characterize the exosomes, we examined the expression levels of exosomal markers, including CD9, CD81 and TSG101, by western blotting. The result showed that both H‐MSC‐exos and N‐MSC‐exos expressed CD91, CD81 and TSG101 (Fig. 2B). We then determined whether these exosomes could be taken up by HUVECs. PKH67 dye was used to stain exosomes. PKH67 fluorescence spots were observed in HUVECs as characterized by immunofluorescence staining (Fig. 1C), indicating an uptake of exosomes by HUVECs. The rate of uptake in the hypoxia group was higher than in the normoxia group (Fig. 1C).

### Exosomes enhance proliferation, migration and tube formation in HUVECs

To explore the promoting potential of exosomes in angiogenesis, we first compared the effect of H‐MSC‐exos and N‐MSC‐exos on the proliferation in HUVECs. CCK‐8 assay showed that exposure of H‐MSC‐exos significantly increased the proliferation of HUVECs compared with N‐MSC‐exos (Fig. 2A). Then we examined the influence of MSC‐exos on HUVEC migration and tube formation, two important steps involved in angiogenesis. The migration assay revealed that the migration ability was significantly higher in the hypoxia group than in the normoxia group (Fig. 2B,C). H‐MSC‐exos displayed a better effect on tube formation of HUVECs compared with N‐MSC‐exos (Fig. 2D,E). Moreover, the expression levels of angiogenesis‐promoting molecules VEGF and CD31 were much higher in H‐MSC‐exo‐treated HUVECs compared with N‐MSC‐exo‐treated HUVECs (Fig. 2F,G). Together, these data indicate that H‐MSC‐exos have better effects on HUVEC proliferation, migration and tube formation than N‐MSC‐exos.

### Hypoxia‐conditioned MSC‐exos promote angiogenesis via induction of HMGB1

**Fig. 3:**
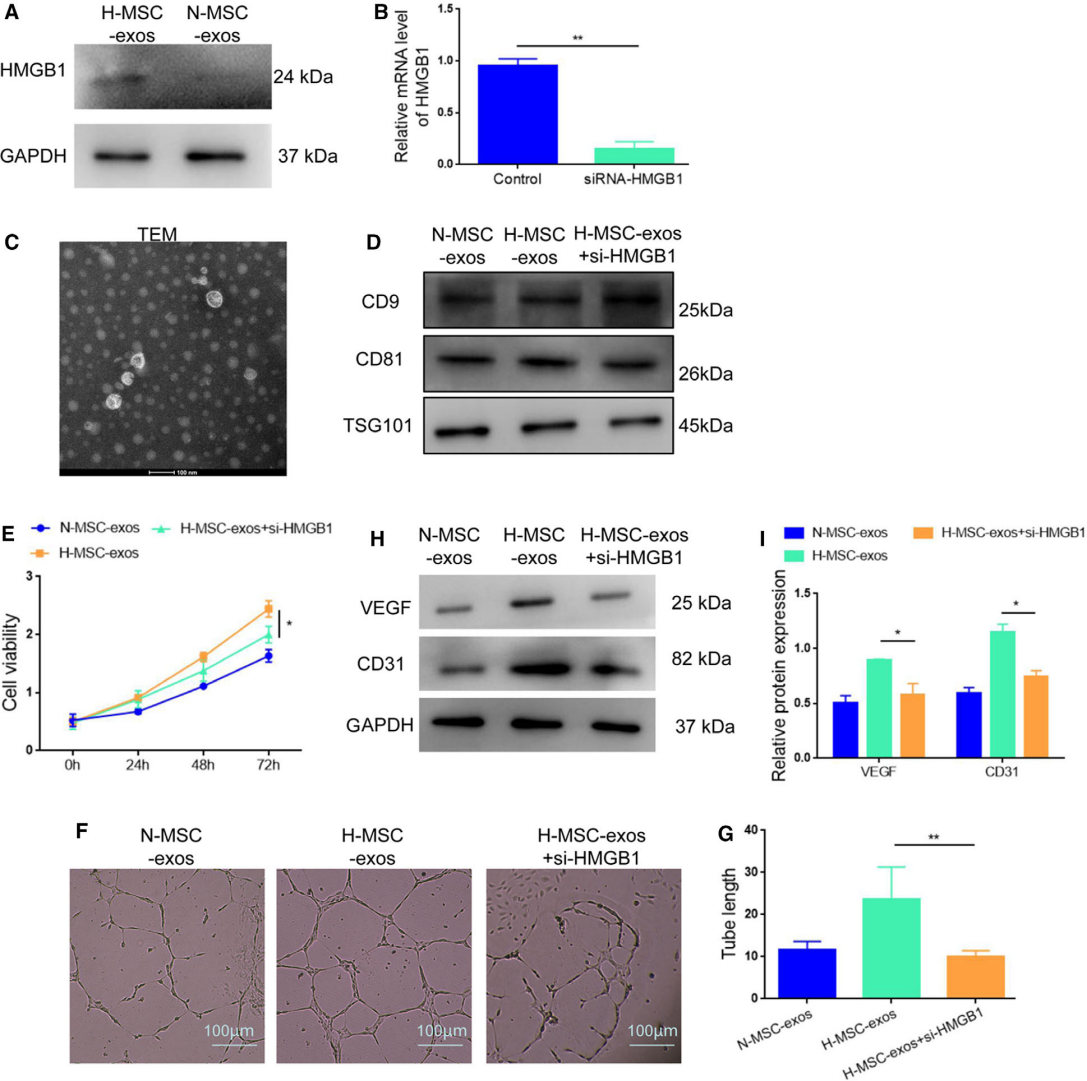
Hypoxic MSC‐derived exosomes enhance angiogenesis through HMGB1. (A) Western blot analysis of HMGB1 expression in HUVECs treated with H‐MSC‐exos (hypoxia/exos) or N‐MSC‐exos (normoxia/exos). (B) Quantitative PCR analysis of HMGB1 expression in HUVECs treated with siRNA. (C) Morphology of hypoxic MSC‐derived exosomes. (D) Western blot analysis of VEGF and CD31 expression in BMSC‐derived exosomes under different conditions. (E) Silencing of HMGB1 by siRNA reduced the viability of HUVECs induced by H‐MSC‐exos. (F, G) Silencing of HMGB1 reduced the tube formation of HUVECs enhanced by H‐MSC‐exos. (H, I) Knockdown of HMGB1 reduced H‐MSC‐exo‐induced VEGF/CD31 expression. *P < 0.05, **P < 0.01; *n* = 3. Data represent the mean ± SD of three separate experiments; comparison was performed with one‐way ANOVA followed by Tukey's *post hoc* test. Scale bars: 100 μm. TEM, transmission electron microscopy.

Next, we explored the molecular mechanism of hypoxic MSC‐exos‐induced angiogenesis and found that the expression of HMGB1 was significantly increased in H‐MSC‐exo‐treated HUVECs compared with N‐MSC‐exo‐treated cells (Fig. 3A). HMGB1 is a proinflammatory cytokine that plays a crucial role in angiogenesis [11, 12]. To confirm the importance of HMGB1 in H‐MSC‐exo‐induced promotion in angiogenesis, we performed siRNA‐mediated knockdown in HUVECs under hypoxic conditions prior to exosome extraction (Fig. 3B–D). CCK‐8 assay showed that silencing of HMGB1 by siRNA remarkably repressed the viability of HUVECs induced by H‐MSC‐exos (Fig. 3E). Tube formation assay also showed that knockdown of HMGB1 abolished the enhancing effect of H‐MSC‐exos on tube formation (Fig. 3F,G). Consistently, the expression levels of VEGF and CD31 increased by H‐MSC‐exos were restrained upon HMGB1 knockdown (Fig. 3H,I). Together, these results suggest that H‐MSC‐exo promotes angiogenesis via up‐regulation of HMGB1.

### Exosomal HMGB1 enhances the expression of hypoxia‐inducible factor‐1α

**Fig. 4:**
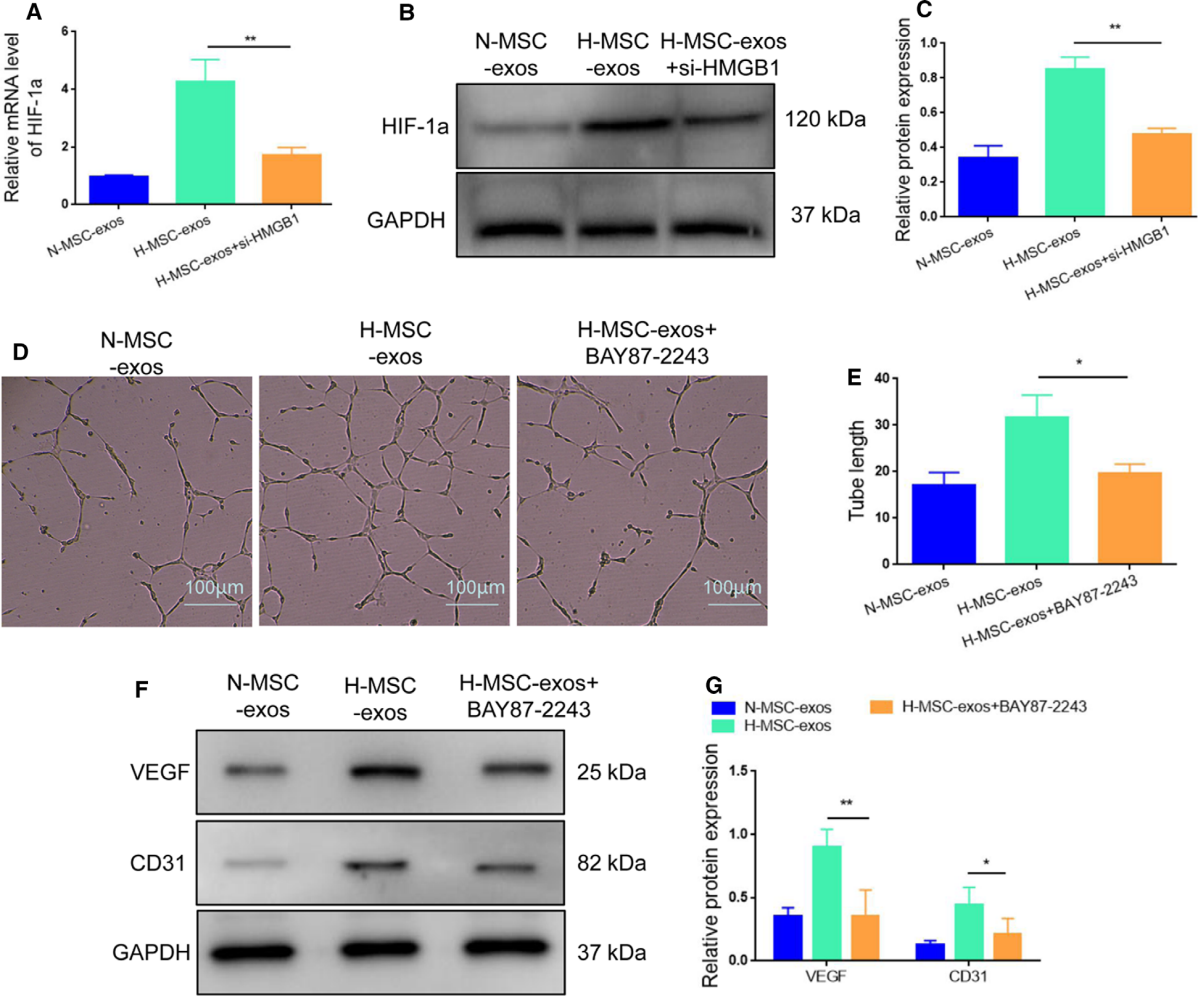
Exosomal HMGB1 up‐regulates HIF‐1α expression. (A–C) Quantitative RT‐PCR and western blot analysis of HIF‐1α mRNA and protein expression in HUVECs under different conditions. (D, E) Treatment with BAY87‐2243 (40 μm) decreased the tube formation of HUVECs enhanced by H‐MSC‐exos. (F, G) Treatment with BAY87‐2243 (40 μm) decreased H‐MSC‐exo‐induced VEGF/CD31 expression. *P < 0.05, **P < 0.01; *n* = 3. Data represent the mean ± SD of three separate experiments; comparison was performed with one‐way ANOVA followed by Tukey's *post hoc* test. Scale bars: 100 μm.

Previous studies report that hypoxia can stimulate hypoxia‐inducible factor‐1a (HIF‐1a), a key upstream regulator of VEGF. Therefore, we speculated that HIF‐1α might contribute to H‐MSC‐exo‐induced angiogenesis. Indeed, treatment with H‐MSC‐exos resulted in a significant increase in the expression of HIF‐1α compared with N‐MSC‐exo exposure (Fig. 4A–C), and this effect of H‐MSC‐exos was blocked by knockdown of HMGB1 (Fig. 4A–C). Administration of HIF‐1a inhibitor, BAY87‐2243, reduced H‐MSC‐exo‐induced tube formation of HUVECs (Fig. 4D,E). Moreover, the expression levels of VEGF and CD31 elevated by H‐MSC‐exos were also decreased upon BAY87‐2243 exposure (Fig. 4F,G). Together, these results suggest that HMGB1‐triggered HIF‐1α activation contributes to H‐MSC‐exos‐induced angiogenesis.

### Exosomal HMGB1 induced angiogenesis via the JNK/HIF‐1α cascade

**Fig. 5:**
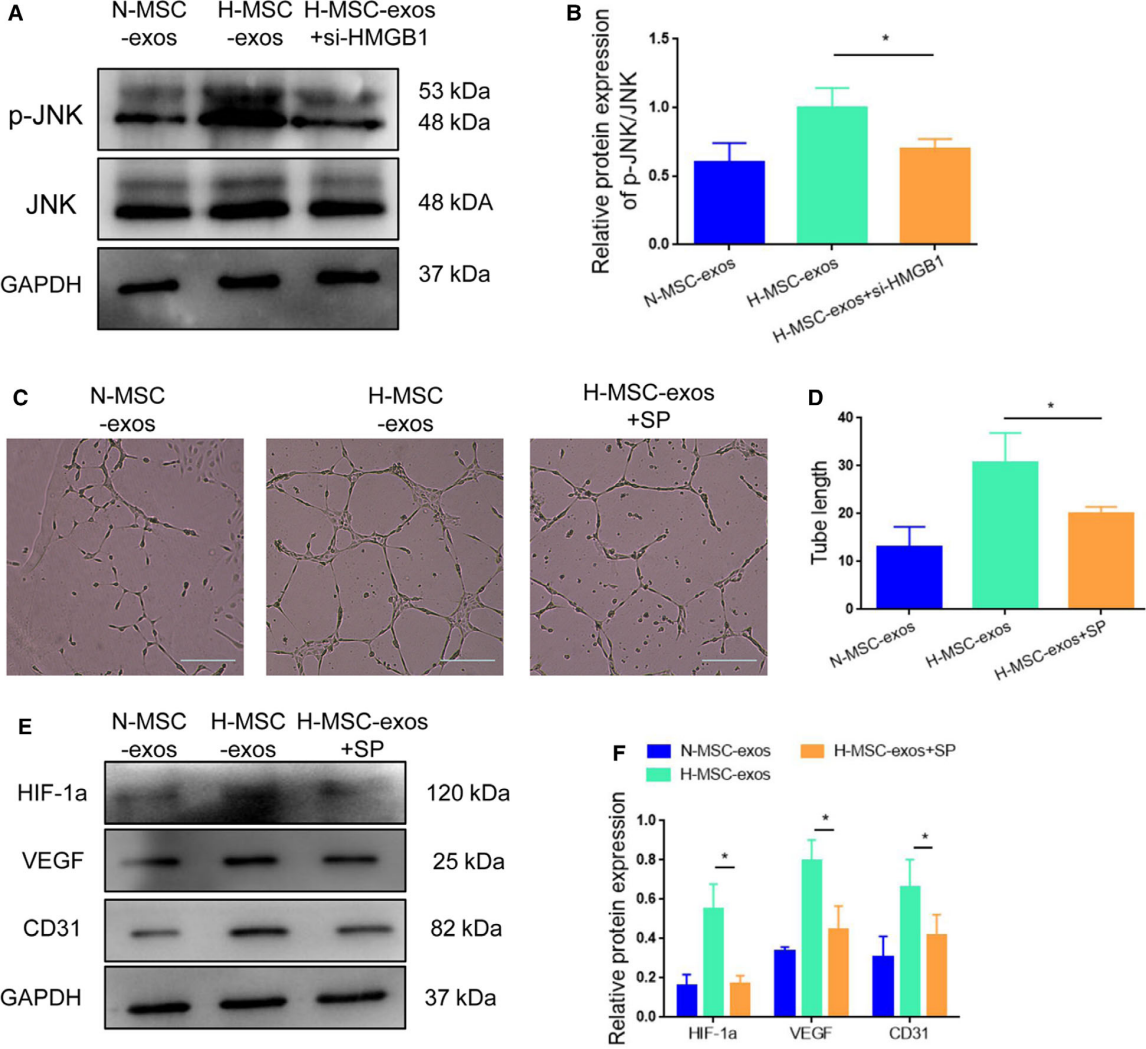
Exosomal HMGB1 promotes angiogenesis via the JNK/HIF‐1α cascade. (A, B) Western blot analysis of JNK expression and phosphorylation in HUVECs under different conditions. (C, D) Administration of SP600125 (60 μm) decreased the tube formation of HUVECs enhanced by H‐MSC‐exos. (E, F) Administration of SP600125 reduced H‐MSC‐exo‐induced VEGF/CD31 expression. *P < 0.05; *n* = 3. Data represent the mean ± SD of three separate experiments; comparison was performed with one‐way ANOVA followed by Tukey's *post hoc* test. Scale bars: 100 μm.

To explore the potential signaling pathway through which exosomal HMGB1 promotes angiogenesis, we examined the protein expression of JNK, an important pathway implicated in angiogenesis. The result showed that application of H‐MSC‐exos in HUVECs increased the phosphorylation of JNK, whereas silencing of HMGB1 by siRNA abolished this effect of H‐MSC‐exos (Fig. 5A,B). Treatment of SP600125 (JNK inhibitor) reduced H‐MSC‐exo‐induced tube formation of HUVECs (Fig. 5C,D). Moreover, SP600125 decreased the levels of HIF‐1α, VEGF and CD1, which were increased by H‐MSC‐exos compared with N‐MSC‐exos (Fig. 5E,F). Collectively, these results suggest that HMGB1 triggers angiogenesis via JNK/HIF‐1α signaling.

## Discussion

Angiogenesis is linked to various processes, such as tissue development, ischemia, inflammation and tissue repair [13]. Accumulating evidence suggests that MSC‐derived exosomes play an important role in angiogenesis, especially under hypoxic conditions [8, 10]. However, the molecular mechanisms are not fully understood. In this study, a series of experiments was performed to verify the promoting effect of exosomes released by MSCs under hypoxic conditions. Moreover, our data reveal that hypoxic‐conditioned MSC‐derived exosomes enhance angiogenesis via regulation of HMGB1.

HMGB1 is a ubiquitously expressed DNA‐binding protein that has multiple functions associated with posttranslational modifications and cellular localization [14]. HMGB1 is involved in cell proliferation, apoptosis, migration, inflammation and invasion [15, 16]. Multiple studies have demonstrated that HMGB1 plays a crucial function in angiogenesis as well. HMGB1 has been shown, using an *in vivo* model, to enhance vessel formation in breast cancer cells [17]. HMGB1 also induces migration, invasion and angiogenesis in ovarian cancer [18]. Leukocyte HMGB1 contributes to vessel remodeling in regenerating muscles [19]. In this study, we observed an up‐regulation of HMGB1 in H‐MSC‐exos compared with the normoxic controls. Silencing of HMGB1 blocked the positive effect of H‐MSC‐exos on cell proliferation and tube formation of HUVECs, indicating the involvement of HMGB1 in H‐MSC‐exos‐induced angiogenesis.

VEGF is considered the most important angiogenesis inducer. HIF‐1α is a key mediator implicated in cellular oxygen homeostasis and facilitates the adaptation to hypoxia [20]. HIF‐1α is closely associated with angiogenesis and is an upstream transcriptional regulator of VEGF [21]. Many studies have reported that under hypoxia conditions, HIF‐1α and VEGF expression levels are increased, which resulted in enhanced cell proliferation, migration and angiogenesis [22, 23, 24, 25]. A previous study has reported that HMGB1 reinforces angiogenesis via induction of HIF‐1α/VEGF in perforated disc cells of the human temporomandibular joint [11]. Our data showed that H‐MSC‐exos had a significantly higher protein level of HIF‐1α and increased expression of VEGF compared with N‐MSC‐exos. Although silencing HMGB1, the elevated expression of HIF‐1α in H‐MSC‐exos was restrained. In addition, treatment with the HIF‐1α inhibitor BAY87‐2243 [26] reduced tube formation of HUVECs. Therefore, our data indicate that H‐MSC‐exos induced angiogenesis via HMGB1/HIF‐1α/VEGF.

The JNK signaling pathway plays a fundamental function in various biological processes, such as cell proliferation, differentiation, survival and migration. Also, the JNK pathway is an important contributor to angiogenesis. It has been reported that HMGB1 induced cell differentiation, apoptosis and migration via the JNK pathway [27, 28]. Moreover, inhibition of JNK signaling has been shown to repress HIF‐1α/VEGF expression [29]. In this study, we found that JNK phosphorylation was increased in H‐MSC‐exos, and this effect was abolished by knockdown of HMGB1. Moreover, administration of the JNK inhibitor SP600125 [26] reduced H‐MSC‐exos‐induced tube formation and the expression of VEGF and CD31, confirming the involvement of JNK signaling in H‐MSC‐exos‐mediated angiogenesis promotion.

## Conclusions

Our findings showed that exosomes released by hypoxic MSCs have stimulative effects on the proliferation, migration and angiogenesis of HUVECs. Hypoxic treatment results in increased expression of HMGB1 in exosomes derived from BMSCs, which activates the JNK pathway and induces HIF‐1α/VEGF expression, consequently enhancing angiogenesis of HUVECs. Hypoxic‐MSC‐derived exosome may represent a novel strategy for the therapy of angiogenesis‐related diseases.

## Conflict of interest

The authors declare no conflict of interest.

## Author contributions

WG and RH designed and performed the experiments, analyzed the data and wrote the manuscript. RH carried out the experiments. JR and WZ performed some of the research. KW helped to review the manuscript. LZ and TL participated in the experimental design, provided financial support and supervised the manuscript. All authors read and approved the final manuscript.

## Data Availability

The data that support the findings of this study are available from the corresponding author upon reasonable request.
